# Estimating the impact of stimulant use on initiation of buprenorphine and extended-release naltrexone in two clinical trials and real-world populations

**DOI:** 10.1186/s13722-023-00364-3

**Published:** 2023-02-14

**Authors:** R. R. Cook, C. Foot, O. A. Arah, K. Humphreys, K. E. Rudolph, S. X. Luo, J. I. Tsui, X. A. Levander, P. T. Korthuis

**Affiliations:** 1 Section of Addiction Medicine, Department of Medicine, Oregon Health & Science University, Sam Jackson Hall, Suite 3370, 3245 SW Pavilion Loop, Portland, OR 97239 USA; 2 Department of Epidemiology, Fielding School of Public Health, University of California, Los Angeles (UCLA), Los Angeles, CA USA; 3 Division of Physical Sciences, Department of Statistics, UCLA College, Los Angeles, CA USA; 4 Research Unit for Epidemiology, Department of Public Health, Aarhus University, Aarhus, Denmark; 5 Center for Innovation to Implementation, VA Palo Alto Health Care System, Palo Alto, CA USA; 6 Department of Psychiatry and Behavioral Sciences, Stanford University, Palo Alto, CA USA; 7 Department of Epidemiology, School of Public Health, Columbia University, New York, NY USA; 8 Division on Substance Use Disorders, Department of Psychiatry, Columbia University, New York, USA; 9 Department of Medicine, University of Washington, Seattle, WA USA

**Keywords:** Stimulants, Methamphetamine, Cocaine, Medications for opioid use disorder, Buprenorphine, Extended-release naltrexone, Transportability, Generalizability

## Abstract

**Background:**

Co-use of stimulants and opioids is rapidly increasing. Randomized clinical trials (RCTs) have established the efficacy of medications for opioid use disorder (MOUD), but stimulant use may decrease the likelihood of initiating MOUD treatment. Furthermore, trial participants may not represent “real-world” populations who would benefit from treatment.

**Methods:**

We conducted a two-stage analysis. First, associations between stimulant use (time-varying urine drug screens for cocaine, methamphetamine, or amphetamines) and initiation of buprenorphine or extended-release naltrexone (XR-NTX) were estimated across two RCTs (CTN-0051 X:BOT and CTN-0067 CHOICES) using adjusted Cox regression models. Second, results were generalized to three target populations who would benefit from MOUD: Housed adults identifying the need for OUD treatment, as characterized by the National Survey on Drug Use and Health (NSDUH); adults entering OUD treatment, as characterized by Treatment Episodes Dataset (TEDS); and adults living in rural regions of the U.S. with high rates of injection drug use, as characterized by the Rural Opioids Initiative (ROI). Generalizability analyses adjusted for differences in demographic characteristics, substance use, housing status, and depression between RCT and target populations using inverse probability of selection weighting.

**Results:**

Analyses included 673 clinical trial participants, 139 NSDUH respondents (weighted to represent 661,650 people), 71,751 TEDS treatment episodes, and 1,933 ROI participants. The majority were aged 30–49 years, male, and non-Hispanic White. In RCTs, stimulant use reduced the likelihood of MOUD initiation by 32% (adjusted HR [aHR] = 0.68, 95% CI 0.49–0.94, *p* = 0.019). Stimulant use associations were slightly attenuated and non-significant among housed adults needing treatment (25% reduction, aHR = 0.75, 0.48–1.18, *p* = 0.215) and adults entering OUD treatment (28% reduction, aHR = 0.72, 0.51–1.01, *p* = 0.061). The association was more pronounced, but still non-significant among rural people injecting drugs (39% reduction, aHR = 0.61, 0.35–1.06, *p* = 0.081). Stimulant use had a larger negative impact on XR-NTX initiation compared to buprenorphine, especially in the rural population (76% reduction, aHR = 0.24, 0.08–0.69, *p* = 0.008).

**Conclusions:**

Stimulant use is a barrier to buprenorphine or XR-NTX initiation in clinical trials and real-world populations that would benefit from OUD treatment. Interventions to address stimulant use among patients with OUD are urgently needed, especially among rural people injecting drugs, who already suffer from limited access to MOUD.

**Supplementary Information:**

The online version contains supplementary material available at 10.1186/s13722-023-00364-3.

## Introduction

Concomitant use of opioids and stimulants, primarily methamphetamine and cocaine, increased by over 80% in the past decade [1–3], contributing to an overdose epidemic that killed over 100,000 Americans in 2021 [4]. While overdose deaths due to prescription opioids and heroin are declining, use of and mortality from highly potent synthetic drugs such as fentanyl and methamphetamine (MA) are steeply rising [4–6]. Co-use of opioids and cocaine, especially co-injection (“speedballs”), are also associated with increased risk of overdose [7]. Medications for opioid use disorder (MOUD), including methadone, buprenorphine, and extended-release naltrexone (XR-NTX), are effective treatments for reducing opioid use, achieving remission from OUD, preventing overdose, and saving lives [8–10].

However, concomitant stimulant use may reduce rates of MOUD initiation [11, 12], inpatient and outpatient opioid treatment engagement, and retention in treatment [13]. Stimulant use may also be associated with decreased opioid abstinence during MOUD treatment, although results are mixed and may depend on type of stimulant [13–15]. In addition, most of these data come from retrospective reviews of medical records, which have low sensitivity to detect stimulant use [16], or studies with small samples and substantial confounding concerns [13]. As the prevalence of co-use of opioids with MA or cocaine increases [3, 17], overdose deaths involving stimulants and opioids follow. Methodologically robust, novel strategies to understand the role of stimulant use in opioid treatment initiation, engagement, and outcomes remain urgently needed [18].

Randomized clinical trials (RCTs) typically collect extensive covariate data that investigators can use to better control bias (i.e., increase internal validity) when investigating nonrandomized secondary questions. However, RCT samples are usually not representative of the larger populations intended to receive treatments under study [19], threatening external validity, and RCTs of treatments for substance use disorders (SUDs) are no exception [20]. SUD treatment trial samples tend to differ from real-world SUD patient populations on age [20], sex [21, 22], race [21, 22], education [23], income [21, 24, 25], and SUD severity [21, 23–26], among other dimensions. People living in rural areas, who already suffer from limited access to medical care including SUD treatment [27], are also underrepresented in RCTs [28]. Further, trials may limit enrollment to participants with a single SUD or fail to recruit representative patients with multiple SUDs, limiting the ability to answer questions about typical clinical patients who use multiple substances [29]. Conversely, large-scale “real-world” data sources may be representative, but often lack the data elements, validity, or specificity to identify and estimate unbiased exposure effects [30]. In particular, few SUD RCTs are implemented to test the real-world effectiveness of SUD treatment. The National Drug Abuse Treatment Clinical Trials Network (CTN) is the *only* government sponsored entity in the US that carries out large-scale pragmatic SUD treatment clinical trials, and the large datasets collected within the CTN contain standardized data elements and similarities in design that presented an opportunity to study SUD treatment in a broader context.

Transportability analysis is a novel analytic solution to this problem, formally defining when and how statistical estimates may be extrapolated from a given source population to a different target population [31–33]. When transportability analysis is conducted with nested populations, i.e., the source population is a subset of the target, it is called generalizability analysis. In a generalizability analysis, detailed exposure, outcome, and covariate data from an RCT can be fused with broad, representative data sources to support the projection of the exposure effect from the RCT onto a real-world target population of interest. The theoretical framework and assumptions underlying transportability/generalizability analysis are well-described [31–33]. We use generalizability analysis to estimate the association between stimulant use and MOUD initiation in three real-world populations of interest. This technique allows us to leverage the strengths of narrowly focused, precise clinical trials and broad, widely representative datasets to answer questions each data source independently cannot.

**Fig. 1:**
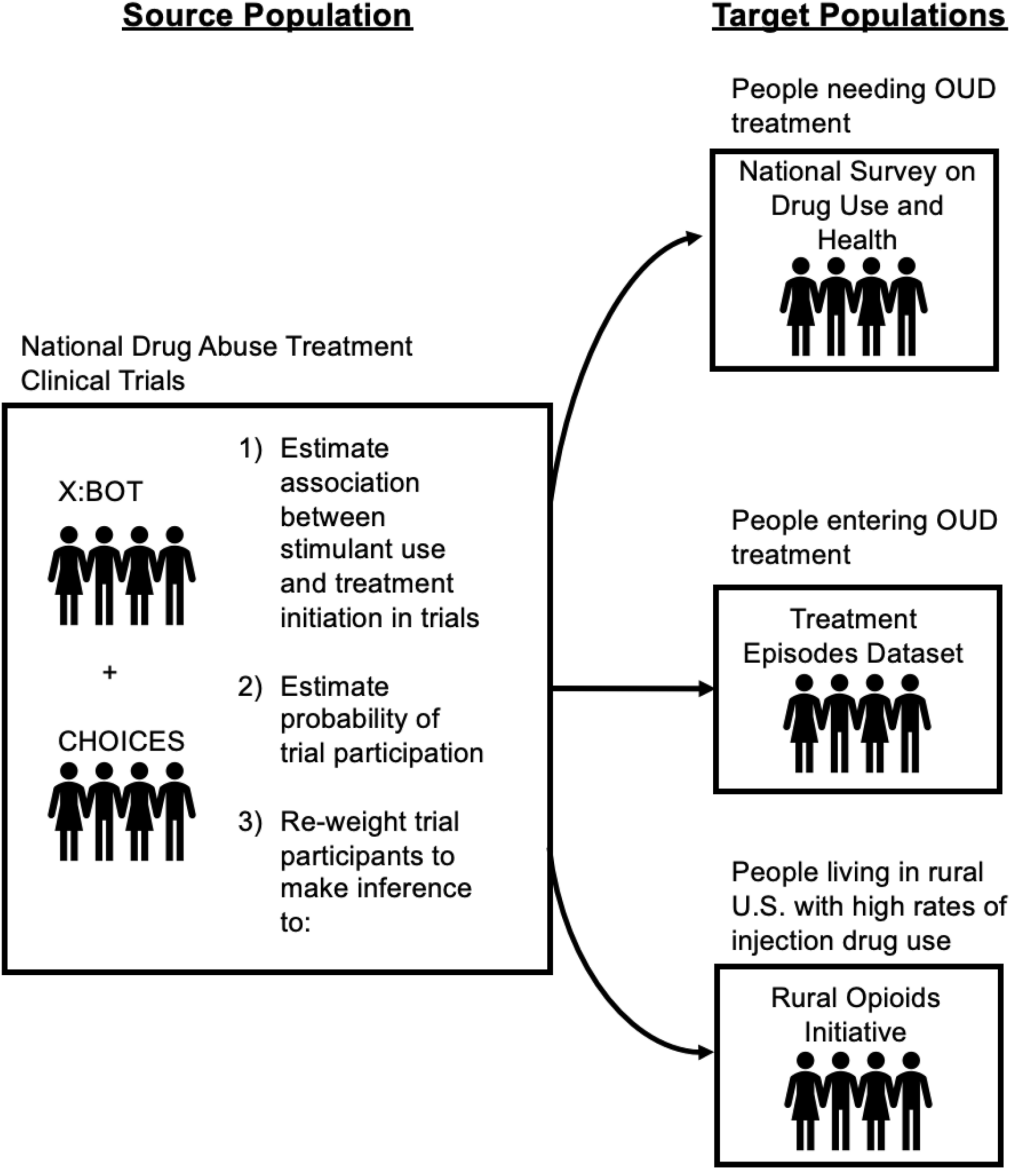
Study overview. Data from the source population, two National Drug Abuse Treatment Clinical Trials Network studies, is pooled to estimate the association between stimulant use and initiation of medication for opioid use disorder in the trials. Differences between trial participants and target populations are used to estimate the probability of trial participation. Inference is made in target populations by re-weighting trial participants to better match the characteristics of individuals in target populations

The primary aim of this study is to estimate the association between stimulant use and initiation of buprenorphine or XR-NTX for the treatment of OUD. To complete this aim, we conducted a two-part analysis. First, we estimated associations in pooled data of two NIDA-CTN clinical trials. Second, we generalized results to three target populations of interest: (1) civilian, noninstitutionalized, housed adults identifying the need for OUD treatment, as characterized by the National Survey on Drug Use and Health (NSDUH); (2) adults entering OUD treatment, as characterized by Treatment Episodes Dataset (TEDS); and (3) adults with opioid misuse and high rates of injection drug use (IDU) living in rural regions of the U.S., as characterized by the Rural Opioids Initiative cohort (ROI). Figure 1 provides a visual depiction of our study aims and procedures. We hypothesize that (1) stimulant use reduces the likelihood of initiating MOUD in all populations, and (2) the magnitude of this reduction is larger in real-world populations of adults with opioid misuse than in highly-selected trial participants.

## Methods

### Data sources and populations

The source (or study) population for our analyses was the pooled study sample of participants in two multisite CTN treatment trials: 0051 (X:BOT [10]; 2014–2017) and 0067 (CHOICES [34]; 2018–2019). Information on each study, including inclusion/exclusion criteria and recruitment and enrollment data are presented in Additional file 1: Table S1. Briefly, X:BOT randomized participants to XR-NTX vs. sublingual buprenorphine in inpatient, medically monitored opioid treatment facilities. CHOICES randomized to XR-NTX vs. treatment as usual in outpatient HIV clinics. However, 93% of treatment-as-usual participants who initiated treatment received buprenorphine; to create a more homogeneous pooled source population, eight CHOICES participants receiving methadone and three receiving only oral naltrexone were excluded. All X:BOT participants were included (corresponding to the original intent-to-treat sample). In both studies, participants provided urine drug screen (UDS) and harmonized questionnaire, and clinical, pharmacy, or laboratory data weekly (X:BOT) or every 4 weeks (CHOICES) for 24 weeks.

We defined three target populations for our analyses. The first is a nationally-representative sample of civilian, housed, noninstitutionalized adults with OUD identifying the need for treatment, as characterized by the NSDUH household 2018 and 2019 survey years [35]. Among all NSDUH respondents, we included those who had OUD, were age 18 or older, and (1) received treatment in the past year, but used nonprescribed opioids in the past month or (2) identified the need for, but had not received, treatment. The second target population was people receiving or planning to receive MOUD treatment, as captured in the 2018 TEDS admissions dataset [36]. Treatment episodes were included if the individual entering treatment met diagnostic criteria for OUD based off the *Diagnostic and Statistical Manual of Mental Disorder—5th edition (DSM-5)*, used heroin or other illicit opioids in the past month, was 18 or older, and had MOUD as part of their intended treatment plan. The third target population was people who inject drugs or misuse opioids in rural areas of the U.S. as characterized by eight sites participating in the ROI [37]. Briefly, the ROI is a consortium of harmonized studies of people who use drugs in rural counties with high overdose rates covering ten states (Illinois, Kentucky, North Carolina, New England [Massachusetts, New Hampshire, Vermont], Ohio, Oregon, West Virginia, Wisconsin). Inclusion criteria varied slightly by study site, but generally, people were eligible for the ROI cohort if they reported IDU or use of opioids to “get high” in the past month. ROI participants were included in this analysis if they were 18 or older and used opioids to get high in the past month. In order to meet positivity assumptions (i.e., to avoid generalizing to people who are strictly excluded from the source population of clinical trials), we also restricted all three target populations to individuals who were English-speaking, not currently pregnant, and without suicidal ideation.

### Exposure, outcome, and covariates

The primary exposure of interest, stimulant use, was defined according to UDS positivity for MA, other amphetamines, or cocaine in clinical trial data. UDS were collected with an FDA-approved one-step temperature-sensitive test cup; a further validity check was performed using a commercially available adulterant test strip. Stimulants are detectable in urine for approximately 3 days to 1 week, depending on dose, route of administration, and type of stimulant [38]. Participants provided UDS samples weekly (X:BOT) or every 4 weeks (CHOICES) and stimulant use was treated as time-varying.

The outcome was time from randomization to MOUD initiation, defined as the date of first prescription of buprenorphine or injection of XR-NTX. Participants who did not initiate MOUD were censored on the last day of study participation.

Demographics, use of other substances, housing status, and depression history from both clinical trial and target population data were included in analyses as covariates as well as in generalizability formulas. Some covariates were measured in different ways across datasets. “Heavy alcohol/benzodiazepine use” was defined as DSM-5 use disorders in the clinical trials and the NSDUH. In TEDS, heavy use was defined by the appearance of benzodiazepines or alcohol on the list of reasons for entering treatment. In the ROI, use of benzodiazepine or alcohol 15 or more days in the past month was classified as heavy use. Injection drug use was past 30 days in clinical trials, TEDS, and ROI, and the past year in NSDUH. Experience of recent homelessness was the past month in the CTN trials, past 6 months in the ROI, current in TEDS, and not measured in the NSDUH household survey. Depression history was not measured in TEDS or ROI. Some covariate data was missing in all sources: 6% of cases from the clinical trials contained missing data, 2% of NSDUH surveys, 9% of TEDS episodes, and 3% of ROI surveys. Missing covariate data were multiply imputed using chained equations [39].

### Statistical analyses

#### Creation of a selection diagram

Prior to analyses, a causal “selection diagram” (Additional file 1: Fig. S1) was created by the study team using a combination of subject-matter expertise, protocols, previous literature, and data. Selection diagrams assist investigators who conduct transportability analyses in identifying variables that modify the effect of the exposure (stimulant use) and differ in distribution between source and target populations. All such variables must be included in transportability analyses to identify valid transportable exposure effects [33]. In addition, in an observational setting (i.e., stimulant use is not randomized), the selection diagram is used to identify important covariates to include in outcome models. Trial protocols informed the selection diagrams by explicitly outlining inclusion and exclusion criteria for each trial. Subject matter expertise and prior literature were used to identify determinants of study outcomes (e.g., factors that influence or are associated with MOUD initiation other than through stimulant use) and thus potential key variables that may modify the effect of stimulant use on the outcome. Authors with expertise in addiction medicine reviewed selection diagrams to assess their completeness and appropriateness. To describe differences between CTN trial samples and the three target populations, we calculated standardized mean differences between CTN trial samples and each target population for all variables included in the transportability analyses.

#### Stage 1: estimation of stimulant use associations in the clinical trials

Among participants in the clinical trials, cumulative incidences of MOUD initiation by baseline stimulant use were described using Kaplan–Meier curves. Cox proportional hazards models were used to estimate associations between time-varying stimulant use and initiation on MOUD. Analyses controlled for CTN trial (X:BOT vs. CHOICES), treatment arm (buprenorphine vs. XR-NTX), age, sex, race, education, employment, depression history, past 30-day homelessness, alcohol use disorder, past 30-day IDU, and time-varying opioid and benzodiazepine use (we focused on benzodiazepines specifically owing to their increasing co-involvement in opioid overdose deaths [40]). Analyses were repeated, stratified by type of MOUD (i.e., buprenorphine and XR-NTX), controlling for the same covariate set. To be able to continue to stage 2 and generalize results from these pooled clinical trials conducted in heterogeneous populations, we assume a common effect of stimulant use on MOUD initiation (conditional on measured covariates) across populations. We tested this assumption by fitting a model with an interaction between stimulant use and trial. We also tested this explicitly using a binary outcome of initiation [41]. Neither test was statistically significant (*p* > 0.3 for both), supporting that our assumption was reasonable.

#### Stage 2: estimation of transported associations in real-world populations

Using variables identified in the selection diagrams, we calculated stabilized inverse probability of selection weights (IPSW) [42]. The probability of selection into the CTN trials was calculated conditional on selection variables (Table 1; Additional file 1: Fig. S1) using logistic regression models. Probabilities were averaged across 10 datasets generated with multiple imputation of covariate data. Three sets of weights were generated, one for each target population (NSDUH, TEDS, ROI). Additional file 1: Table S2 details the three sets of IPSW that were used in the transportability analyses. In the analysis of NSDUH data, the IPSW model was calculated incorporating sampling weights to account for the complex survey design [43]. Each set of weights was trimmed at the 1st and 99th percentiles, as is generally recommended in inverse probability weighting procedures to balance the bias-variance tradeoff due to extreme observations [44, 45]. Additional file 1: Table S3 presents descriptive statistics and shows bias and relative precision of generalized stimulant use associations using trimmed vs. untrimmed IPSW.

**Table 1:** Characteristics and behaviors of participants in CTN clinical trials compared to people with opioid use disorder (1) identifying need for treatment [National Survey on Drug use and Health (NSDUH)], (2) entering substance use treatment [Treatment Episodes Dataset (TEDS)], and (3) from rural regions of the U.S. [Rural Opioids Initiative (ROI)]

Characteristic/ behavior	CTN trials N = 673 N (%)	NSDUH^a^ N = 661,650 N (%)	SMD	TEDS N = 71,751 N (%)	SMD	ROI N = 1933 N (%)	SMD
Age			0.085		0.16		0.19
18–29	242 (36.0)	229,715 (34.7)		20,430 (28.5)		593 (31)	
30–49	333 (49.5)	314,933 (47.6)		39,559 (55.1)		1118 (58.5)	
50+	98 (14.6)	117,002 (17.7)		11,762 (16.4)		199 (10.4)	
Male sex	465 (69.1)	361,130 (54.6)	0.30	42,805 (59.7)	0.2	1143 (59.1)	0.21
Race/ethnicity			0.35		0.28		0.74
Non-Hispanic Black/African-American	117 (17.4)	65,584 (9.9)		9720 (13.5)		56 (2.9)	
Non-Hispanic White	425 (63.2)	520,682 (78.7)		51,373 (71.6)		1591 (82.3)	
Hispanic/Latino	109 (16.2)	65,379 (9.9)		6452 (9)		77 (4)	
Other	22 (3.3)	10,004 (1.5)		4206 (5.9)		209 (10.8)	
Education			0.26		0.36		0.35
< High school	171 (25.9)	108,560 (16.4)		17,366 (25.3)		437 (22.6)	
High school diploma/GED	225 (34.1)	229,536 (34.7)		34,031 (49.5)		945 (48.9)	
Some college	220 (32.4)	256,390 (38.8)		14,152 (20.6)		507 (26.3)	
Bachelor’s or higher	43 (6.5)	67,164 (10.2)		3187 (4.6)		42 (2.2)	
Employed	190 (24.6)	284,520 (43)	0.40	18,563 (22)	0.06	765 (39.6)	0.32
Homeless	185 (27.5)	N/A		7532 (11^b^)	0.43	1015 (53.3^b^)	0.55
Depression history	230 (34.2)	286,791 (44.0^b^)	0.20	N/A		N/A	
Heavy alcohol use	73 (10.8)	77,006 (11.6)	0.025	5876 (8.2)	0.09	240 (12.4)	0.05
Heavy benzodiazepine use	65 (9.7)	105,825 (16)	0.19	5869 (8.2)	0.05	156 (8.1)	0.06
Injection drug use	443 (65.5)	240,535 (36.4)	0.63	38,839 (54.1)	0.26	1851 (95.8)	0.81
Average SMD			0.27		0.21		0.36

*CTN* National Drug Abuse Treatment Clinical Trials Network, *NSDUH *National Survey on Drug Use and Health, *TEDS* Treatment Episodes Dataset, *ROI* Rural Opioids Initiative, *SMD* standardized mean difference

^a^Weighted N (%). Actual N = 139

^b^Denominator excludes missing data in depression and homelessness variables

To complete the transportability analyses, we re-fit Cox regression models incorporating IPSW with robust standard errors (sandwich estimator) [42]. By incorporating the weights, these models estimate the association between stimulant use and MOUD initiation in the three target populations. As detailed above, the same covariate sets were used for confounder adjustment (“Stage 1: estimation of stimulant use associations in the clinical trials”). As in the clinical trials, we first present overall estimates, and then estimates stratified by type of MOUD. All analyses were conducted in R v.4.0.5 with the ‘mice’, ‘survival’, ‘emmeans’, and ‘ggplot2’ packages.

## Results

### Characteristics of RCT versus real-world target populations

This analysis included 673 clinical trial participants (n = 570 X:BOT and n = 103 CHOICES), 139 NSDUH respondents (weighted to represent 661,650 people), 71,751 TEDS treatment episodes, and 1933 ROI participants. Table 1 details the characteristics of the CTN trial participants and the three target populations. The majority of all included participants/respondents were between the ages of 30–49, male, and non-Hispanic White. Most had achieved a high school diploma or GED or completed some college, but fewer than half were employed. The age distribution was similar between clinical trial and target populations, but trial participants were substantially more likely to be male and Non-Hispanic Black or Hispanic than all target populations. There were differences in education levels, with ROI and TEDS participants generally having the lowest levels of formal education, clinical trial participants in the middle, and NSDUH respondents having the highest levels of education. Employment rates were about half as high among clinical trial participants and TEDS patients compared to the NSDUH and ROI samples. About a third of trial participants had a history of depression compared to 44% of the NSDUH sample (TEDS and ROI datasets did not contain depression data). Of ROI participants, 53% reported experiencing homelessness compared to 27% of clinical trial participants and 11% of the TEDS sample. Around 10% of each population used alcohol heavily. Rates of heavy benzodiazepine use were also around 10%, except for 16% in the NSDUH. IDU rates varied widely between populations: Two-thirds of clinical trial participants reported recently injecting drugs compared to 96% of ROI participants, 36% of the NSDUH respondents, and 54% of the TEDS sample. Overall, the clinical trial population was most like the TEDS population (average SMD = 0.21), less similar to the NSDUH respondents (average SMD = 0.27), and least so to the ROI participants (average SMD = 0.36).

### Rates of stimulant use and treatment initiation or engagement

All individuals in the CTN studies had access to and were expected to initiate buprenorphine or XR-NTX as part of their study participation. A total of 80% of participants initiated MOUD during the trials (543/673). Rates were higher in X:BOT (83%) than CHOICES (68%), explained by their inpatient vs. outpatient treatment initiation settings. Initiation on buprenorphine was more likely than XR-NTX (90% vs. 71%). Similarly, all included TEDS treatment episodes had MOUD as part of the treatment plan, but TEDS does not report whether or not MOUD treatment was actually initiated. The NSDUH does not ask about MOUD treatment specifically, but 58% of included NSDUH respondents accessed some form of SUD treatment in the past year (but were still actively using opioids at the time of survey response). Among those who had not, 45% reported trying but being unable to access treatment and 55% had not tried. Among ROI participants, 38% reported engagement in inpatient or outpatient SUD treatment in the past 6 months and 64% of those without recent treatment engagement indicated that they had tried but were unable to obtain treatment.

At baseline, 105/673 (16%) CTN trial participants tested UDS positive for stimulants (26 MA/amphetamines only, 75 cocaine only, and four used both). Monthly stimulant use rates at follow-up ranged from 15 to 30% (mean 25%, standard deviation 5%).

### Stage 1: associations between stimulant use and MOUD initiation in the clinical trials

**Fig. 2:**
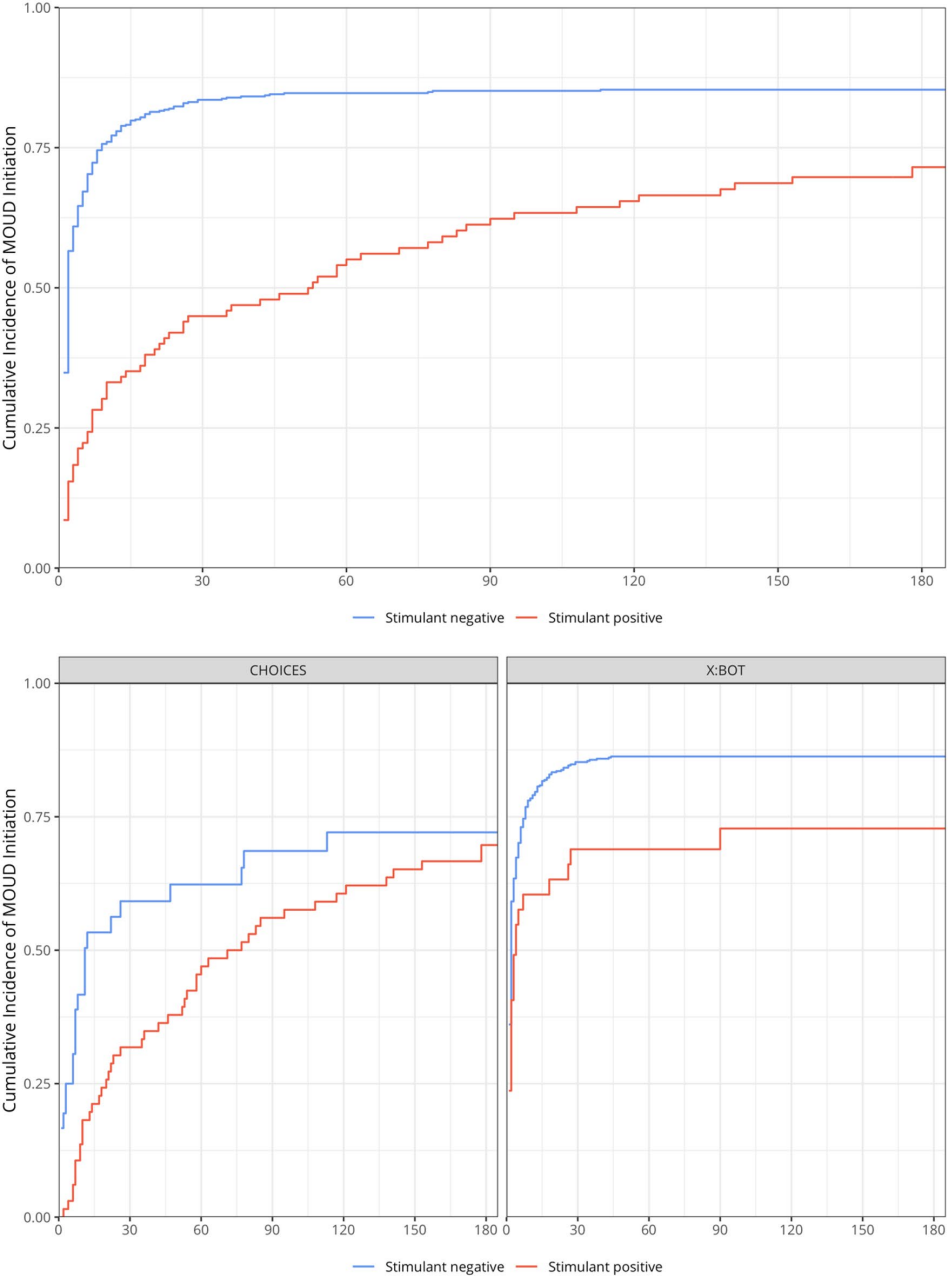
Cumulative incidence of medication for opioid use disorder (MOUD) initiation by baseline stimulant use overall and stratified by CTN study (N = 673; n = 570 X:BOT and n = 103 CHOICES)

Figure 2 displays the cumulative incidence of MOUD initiation in each CTN trial. Although fewer CHOICES participants initiated MOUD overall, there was little evidence that the associations between stimulant use and MOUD initiation differed by trial (*p* value for interaction = 0.884). In an analysis of the CTN trials, stimulant use reduced the likelihood of MOUD initiation by 32% (adjusted HR [aHR] = 0.68, 95% CI 0.49 to 0.94, *p* = 0.019). The impact of stimulant use on XR-NTX was larger (51% reduction, aHR = 0.49, 95% CI 0.28 to 0.86, *p* = 0.013) than on buprenorphine (33% reduction, aHR = 0.67, 95% CI 0.48 to 0.92, *p* = 0.015).

### Stage 2: associations between stimulant use and MOUD initiation in the target populations

**Fig. 3:**
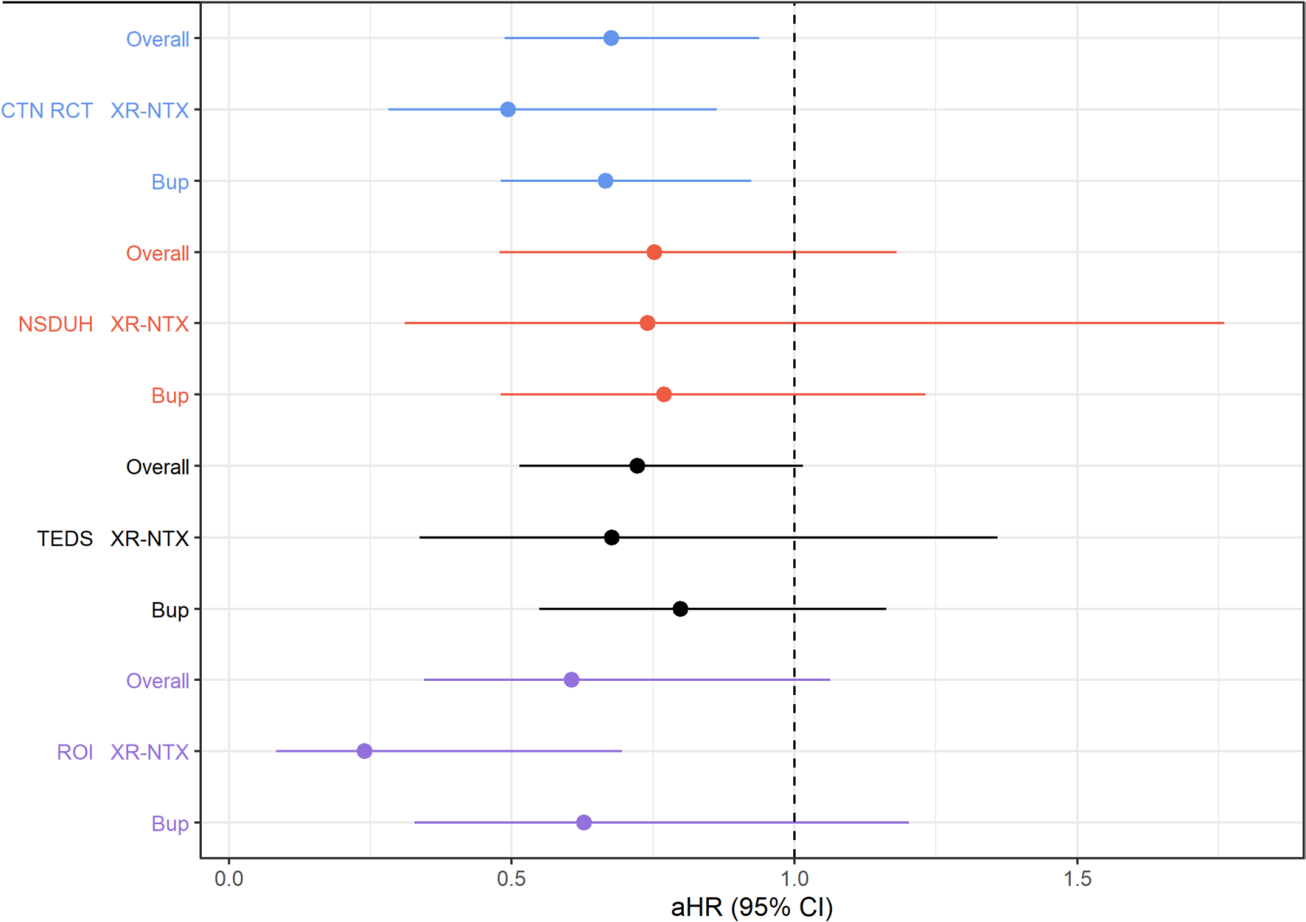
Associations between stimulant use and initiation of buprenorphine or extended-release naltrexone. Results from randomized controlled trials are generalized to target populations with opioid misuse. *MOUD *medication for opioid use disorder, *CTN RCT *National Drug Abuse Treatment Clinical Trials Network, *NSDUH *National Survey on Drug Use and Health, *TEDS *Treatment Episodes Dataset, *ROI *Rural Opioids Initiative, *XR-NTX *extended-release naltrexone, *Bup *buprenorphine, *aHR *adjusted hazard ratio

Table 2; Fig. 3 summarize results of our generalizability analyses. When adjusting for differences in demographics, substance use behaviors, and depression history between clinical trial participants and people needing treatment, as characterized by the NSDUH population, stimulant use reduced the likelihood of MOUD initiation by 25% overall (aHR = 0.75, 95% CI 0.48 to 1.18, *p* = 0.215). Estimates were similar when examining buprenorphine (23% reduction, aHR = 0.77, 0.48 to 1.23, *p* = 0.275) and XR-NTX independently (26% reduction, aHR = 0.74, 0.31 to 1.76, *p* = 0.496). However, all estimates of association displayed substantial uncertainty and none met the threshold for statistical significance.

**Table 2:** Associations between stimulant use and initiation on medication for opioid use disorder

Population	MOUD	aHR (95% CI)	*p*	Relative risk reduction (%)
CTN trials	Overall	0.68 (0.49, 0.94)	0.019	32
	Buprenorphine	0.67 (0.48, 0.92)	0.015	33
	XR-NTX	0.49 (0.28, 0.86)	0.013	51
NSDUH	Overall	0.75 (0.48, 1.18)	0.215	25
	Buprenorphine	0.77 (0.48, 1.23)	0.275	23
	XR-NTX	0.74 (0.31, 1.76)	0.496	26
TEDS	Overall	0.72 (0.51, 1.01)	0.061	28
	Buprenorphine	0.80 (0.55, 1.16)	0.241	20
	XR-NTX	0.68 (0.34, 1.36)	0.273	32
ROI	Overall	0.61 (0.35, 1.06)	0.081	39
	Buprenorphine	0.63 (0.33, 1.20)	0.161	37
	XR-NTX	0.24 (0.08, 0.69)	0.008	76

*CTN* National Drug Abuse Treatment Clinical Trials Network, *NSDUH *National Survey on Drug Use and Health, *TEDS *Treatment Episodes Dataset, *ROI *Rural Opioids Initiative, *XR-NTX *extended-release naltrexone, *aHR *adjusted hazard ratio

Adjustment for differences between clinical trial participants and individuals entering treatment, as captured in the TEDS admissions dataset, suggested little change in the point estimate of stimulant use as observed in the trials (aHR = 0.72, 95% CI 0.51 to 1.01, *p* = 0.061). Medication-specific effect sizes in this population were attenuated compared to the trials, but similarly larger for XR-NTX (32% reduction, aHR = 0.68, 95% CI 0.34 to 1.06, *p* = 0.273) than buprenorphine (20% reduction, aHR = 0.80, 95% CI 0.55 to 1.16, *p* = 0.241), although confidence intervals were large.

Transportability analyses suggested a larger impact of stimulant use on MOUD initiation among rural people with high rates of IDU as compared to the clinical trials; we estimate that people using stimulants would be 39% less likely than non-users to initiate MOUD in this population (aHR = 0.61, 95% CI 0.35 to 1.06, *p* = 0.081). Additionally, larger differences were noted by type of MOUD: Stimulant use reduced the likelihood of XR-NTX initiation by as much as 76% (aHR = 0.24, 95% CI 0.08 to 0.69, *p* = 0.008), whereas a reduction of 37% was estimated for buprenorphine (aHR = 0.63, 95% CI 0.33 to 1.20, *p* = 0.161).

## Discussion

Our study suggests that stimulant use is a barrier to buprenorphine or XR-NTX initiation. Whether associations were estimated in randomized trials or generalized to people needing SUD treatment, people entering SUD treatment, or people from rural communities with high rates of IDU, concurrent stimulant use was associated with a 25–40% reduction in the likelihood of initiating MOUD. Although many of our generalized results failed to reach statistical significance, previous research has generally shown a negative association between stimulant use, especially MA, and MOUD initiation, with effect sizes similar to those observed in our study [11–13, 46, 47]. However, most of these studies have been cross-sectional, retrospective chart reviews, or included stimulant use as a self-reported, time-fixed exposure. By grounding our generalizability analysis in rich clinical trials data, our work adds valuable evidence to support the importance of addressing stimulant use among people with OUD seen in different settings and the need to develop strategies to improve MOUD initiation, such as outreach to needle exchanges or communities of people experiencing homelessness, given the higher rates of overdose and mortality in those with concomitant stimulant use.

Understanding differences between source (i.e., clinical trial participants) and target populations (people identifying the need for treatment, people entering treatment, and rural people with high rates of IDU) is critical to interpreting and applying results. RCTs frequently do not represent real-world populations of clinical relevance, and the degree to which exposure effects estimated from clinical trials are generalizable depends on differences in variables that modify the treatment effect [32]. In this study, we observed that CTN trial participants were most similar to those included in the TEDS dataset, less so to NSDUH respondents, and least so to members of the ROI cohort. Therefore, we also observed that associations between stimulant use and MOUD initiation were more likely to change when generalized to people identifying a need for SUD treatment (NSDUH) and people from rural communities with high rates of IDU (ROI) compared to people entering treatment (TEDS). Likely, a key similarity between CTN and TEDS populations is that both were engaging in SUD treatment. In the trials, participants were extensively supported and encouraged to initiate MOUD as part of their participation, and TEDS treatment episodes were included if MOUD was part of the treatment plan. On the other hand, only about half of NSDUH respondents and 40% of ROI participants had recently engaged in any form of SUD treatment, and many others attempted to, but did not access treatment. Furthermore, the majority of ROI participants were persons who injected drugs, who may have more severe addiction and would be less likely to engage in treatment.

Stimulant use may inhibit MOUD initiation through several causal pathways, both structural and personal. Historically, clinical guidelines discouraged buprenorphine treatment for people who use other substances along with opioids; these were updated in 2020 with the caveat that this population may require additional support and more intensive care [48, 49]. Stigma from providers, communities, and other people who use drugs remains a substantial barrier to medication treatment engagement among people using both opioids and stimulants [50, 51]. Stimulant use may also affect one’s motivation, resourcefulness, social network, or physical and psychological capability to initiate MOUD [52], which may be differentially affected by access. Yet, although the size of the stimulant use association varied across target populations, it was universally negative, even if many of the generalized estimates were insufficiently precise to be conclusive.

NSDUH respondents were more likely to be white, highly educated, employed, and female than trial participants. The NSDUH is also a household survey, failing to reach the substantial number of people with SUDs who experience homelessness (nearly 30% of clinical trial participants had been recently homeless). Also, rates of IDU, a marker of the severity of substance use disorder, were about half as high in the NSDUH compared to the clinical trials. Results suggest that the impact of stimulant use may have been reduced in this population compared to that observed in the clinical trials, although results failed to reach statistical significance. Differences may be attributable to more support structures, resources, and/or other advantages available to racial/ethnic majority, educated, employed, and housed people with less severe substance use disorders responding to the NSDUH.

The CTN trials seem to be a better approximation of the population entering SUD treatment, as characterized by TEDS. This is supported by a previous analysis of TEDS data showing that people admitted for OUD treatment who also used MA were 35% less likely to have MOUD as part of the treatment plan, an effect size similar to that observed in the trials [17]. Stimulant use still negatively impacted treatment uptake, but estimates of association were similar between TEDS and the clinical trials.

On the other hand, the negative impact of stimulant use on treatment initiation was nearly doubled in the population from rural areas, which were generally non-treatment seeking, had lower levels of education, and much higher rates of homelessness and IDU than the clinical trial participants. Stimulant use may be associated with as much as a 40% reduction in the likelihood of MOUD initiation in this population, which already suffers from limited access to SUD treatment [27], although the result failed to reach statistical significance. However, the negative impact of stimulant use on XR-NTX initiation was especially strong and statistically significant; given limited access to medically-monitored detoxification facilities in rural areas and the challenges people injecting opioids face when initiating a full opioid antagonist [53], our data adds further evidence that the real-world impact of XR-NTX may be limited in this population. Recent data showing extremely high rates of MA use in rural areas [54], rapid eastward spread of MA [55], and westward spread of fentanyl [6] are likely to worsen overdose risk and MOUD initiation for people who use both stimulants and opioids, nationally [56].

Our findings highlight the need for evidence-based interventions to reduce stimulant use among people from rural communities with OUD, who are rarely included in clinical trials [28]. For example, contingency management interventions demonstrated to improve treatment engagement and decrease methamphetamine use in clinical trials, could be adapted and integrated with OUD treatment [57]. While currently no pharmacotherapies are approved for treatment of stimulant use disorders, rigorously studied emerging therapies with modest benefit such as mirtazapine [58] and combined bupropion/extended-release naltrexone for methamphetamine use disorder [59] could be integrated with MOUD treatment, when appropriate. Structural interventions such as peer support services that have been used to improve treatment retention [60], and behavioral therapy (potentially delivered via telehealth) could be adapted to focus on people using both stimulants and opioids.

Generalizability and transportability analyses offer several advantages when answering questions about “real-world” impacts of treatment compared to using a single dataset, even if that dataset is large and representative of the target population [61]. Notably, to conduct generalizability analyses, target population datasets do not always need to contain exposure or outcome information. In this case, the NSDUH did not ask about MOUD treatment until 2019, and even then, in a single question. The NSDUH and ROI are cross-sectional surveys and substance use data, including use of opioids and stimulants, are not longitudinal. TEDS admission data does not confirm MOUD receipt or collect quantitative substance use data. Therefore, the ability to answer our study question of interest in these datasets alone would be very limited. Additionally, secondary analyses of data not collected for research purposes may be especially subject to bias. For example, large national studies of VA electronic health record data [62] and private insurance claims data [63] showed small but positive associations between stimulant use and receipt of MOUD (RR ~ 1.2–1.3), in contrast to several other studies finding the opposite. On the other hand, data collected in clinical trials usually have higher validity; in these CTN studies, substance use data were collected via a biomarker in a time-varying fashion and the exact day a participant initiated MOUD was recorded. Thus, even though clinical trials are usually incompletely representative, the extensive datasets allow for less biased inference about the relationship between exposure and outcome, even if the exposure under study was not randomized. Generalizability analysis allows one to combine these strengths with broader, more representative data sources to answer questions about treatment effects in real-world populations of interest—something that each dataset alone could not.

Our study is subject to several limitations. Most notably, generalizability analysis relies on untestable assumptions about the causal mechanisms giving rise to differential treatment effects across populations. All observational studies rely on similar untestable assumptions; we utilized a combination of subject-matter expertise, protocols, previous literature, and data to select a covariate set for IPSW estimation, and we publish our assumptions in the form of a “selection diagram” (Additional file 1: Fig. S1). However, not all key variables were available in the data; we identify two missing items, engagement in general medical care (e.g., primary care) and psychiatric comorbidities other than depression, in the diagram. There are likely other unidentified effect-modifying variables that we were unable to account for. Second, the ability to transport to a target population can be limited by the available data characterizing differences between the study and target populations. For example, the NSDUH has been specifically criticized for underrepresenting injection drug use [64], a key modifier of our findings. The NSDUH also relies on complex survey weighting; results are subject to higher uncertainty and may be strongly influenced by a select few heavily-weighted individuals. TEDS data is primarily comprised of SUD treatment episodes in facilities receiving public funding, but the scope of facilities and medical records included varies considerably by state. Thus, TEDS data may not reflect the experiences of all people entering substance use treatment [65]. Most inference made with these data would be subject to these challenges; they are not specific to generalizability analyses. The assumption of positivity, essentially, that there is some amount of overlap in selection characteristics between source and target populations, is seldom met in finite samples. Although we are unable to definitively conclude that positivity was sufficiently met, we excluded strict positivity violations (e.g., pregnant people who were excluded from both clinical trials) and explored the impact of positivity violations through weight trimming (Additional file 1: Table S3), consistent with best practices [44, 45]. Estimators of generalized treatment effects vary in efficiency, with weighting-based strategies being relatively inefficient, leading to wide confidence intervals and possibly explaining lack of statistical significance. Unfortunately, to the best of our knowledge, more efficient estimators (g-computation, targeted maximum likelihood based-methods) have not yet been developed for time-to-event outcomes with time-varying covariates. Also, we were unable to split stimulant use into MA/amphetamines and cocaine, due to the relatively small number of MA-positive UDS in the clinical trials. The trials were primarily conducted in East Coast sites, where the prevalence of MA use was lower compared to cocaine during the study periods. Finally, we were unable to examine the impact of stimulant use on methadone treatment, which was not included in X:BOT and only initiated by eight people in CHOICES (whom we excluded). Other studies have examined the impact of stimulants on methadone initiation [13].

## Conclusion

This study emphasizes the negative impact that stimulant use may have on MOUD initiation in trials and extrapolates findings to three real-world populations needing OUD treatment, where polysubstance use is the norm rather than the exception. Although broadening the availability of MOUD is expected to improve the public health burden of opioid use, rapidly increasing rates of concomitant stimulant use may reduce its impact. An analysis of urine drug screens from 150,000 buprenorphine patients showed 10% cocaine and 30% MA positivity rates [66], underscoring the continuing challenge of polysubstance use following MOUD initiation. Efforts to identify efficacious treatments for stimulant use disorders should be intensified and integrated with OUD treatment. Within the changing landscape of the opioid overdose crisis due to fentanyl and other synthetic opioids, which are both rapidly becoming drugs of choice as well as adulterating the stimulant supply, there is greater urgency to address stimulant use among patients with OUD.

## Supplementary Information

**Additional file 1: Figure S1.** Selection diagram fortransportability analysis of the effects of stimulant use on initiation ofmedication for opioid use disorder (MOUD). Variables in gray text were notmeasured in one target population; those in gray boxes were not measured in anytarget population. **Table S1.** Inclusion andexclusion criteria, recruitment, screening and enrollment data for CTN clinicaltrials. **Table S2.** Inverse probabilityof selection weight models. **Table S3.**Distributions of inverse probability of selection weights and bias-variance tradeoffin weight trimming. Bias and precision of the stimulant use estimates arecomputed relative to untrimmed weights.

## Data Availability

CTN 0051 X:BOT data are publicly available on the NIDA datashare website (https://datashare.nida.nih.gov/study/nidactn0051). NSDUH and TEDS are publicily available from the SAMHSA website, https://www.datafiles.samhsa.gov/dataset/national-survey-drug-use-and-health-2019-nsduh-2019-ds0001 and https://www.datafiles.samhsa.gov/dataset/treatment-episode-data-set-admissions-2018-teds-2018-ds0001. CTN 0067 data will be posted to the NIDA datashare website upon de-identification and harmonization; interim requests for data should be directed to the corresponding author (cookry@ohsu.edu). Requests for ROI data should be directed to the data coordinating center (https://ruralopioidinitiative.org/studies.html).

## Abbreviations

MA: Methamphetamine
MOUD: Medications for opioid use disorder
XR-NTX: Extended-release naltrexone
RCT: Randomized controlled trial
SUD: Substance use disorder
CTN: National Drug Abuse Treatment Clinical Trials Network
NSDUH: National Survey on Drug Use and Health
TEDS: Treatment Episodes Dataset
IDU: Injection drug use
ROI: Rural Opioids Initiative
UDS: Urine drug screen
IPSW: Inverse probability of selection weights
