# Supplementary material for: Estimating the impact of stimulant use on initiation of buprenorphine and extended-release naltrexone in two clinical trials and real-world populations
Source: Addict Sci Clin Pract. 2023 Feb 14;18:11. doi: 10.1186/s13722-023-00364-3 (PMC9930351; doi:10.1186/s13722-023-00364-3)
Supplement: Supplementary file 1 — Additional file 1: Figure S1. Selection diagram fortransportability analysis of the effects of stimulant use on initiation ofmedication for opioid use disorder (MOUD). Variables in gray text were notmeasured in one target population; those in gray boxes were not measured in anytarget population. Table S1. Inclusion andexclusion criteria, recruitment, screening and enrollment data for CTN clinicaltrials. Table S2. Inverse probabilityof selection weight models. Table S3.Distributions of inverse probability of selection weights and bias-variance tradeoffin weight trimming. Bias and precision of the stimulant use estimates arecomputed relative to untrimmed weights. [file 13722_2023_364_MOESM1_ESM.docx]

Figure S1. Selection diagram for transportability analysis of the effects of stimulant use on initiation of medication for opioid use disorder (MOUD). Variables in gray text were not measured in one target population; those in gray boxes were not measured in any target population.


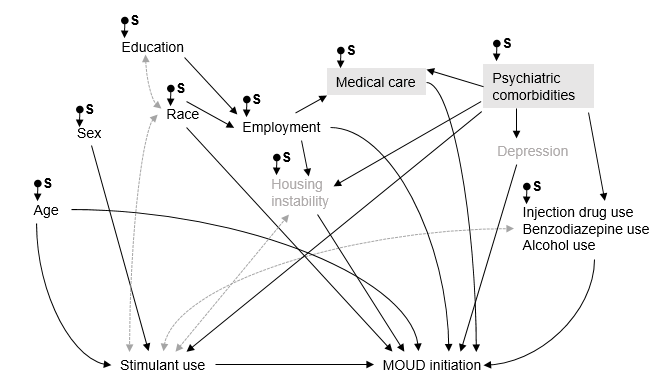


Table S1. Inclusion and exclusion criteria, recruitment, screening and enrollment data for CTN clinical trials

|  | CTN 0051 (XBOT) | CTN 0067 (CHOICES) |
| --- | --- | --- |
| Dates | - Jan 2014 – Jan 2017 | - Feb 2018 – Nov 2019 |
| Inclusion criteria | - At least 18 years old - DSM-5 criteria for opioid-use disorder - Used opioids other than as specifically prescribed within thirty days - Seeking treatment for opioid dependence and willing to accept "agonist-based" or "antagonist-based" therapy - In good-enough general health - Able to provide written informed consent - Able to speak English sufficiently to understand the study procedures - If female of childbearing potential, be willing to practice an effective method of birth control | - At least 18 years old - Participant has provided written informed consent and HIPAA - DSM-5 criteria for moderate/severe opioid-use disorder - Willing to accept "agonist-based" or "antagonist-based" therapy - HIV viral RNA count of > 200 copies/ml - Receiving care or willing to establish ongoing HIV care - If female of childbearing potential, be willing to practice an effective method of birth control |
| Exclusion criteria | - Serious medical, psychiatric or substance use disorder that would make study participation hazardous to the participant - Liver measurements > 5x normal - Suicidal or homicidal ideation - Allergy or sensitivity to study meds - Maintenance on methadone at doses of 30mg or greater - Ongoing pain management with opioids - Pending legal action - Currently pregnant, breastfeeding, or planning on conception - Unable to be safely injected with XR-NTX | - Serious medical, psychiatric or substance use disorder that would make study participation hazardous to the participant - Acutely life-threatening medical illnesses - Severe, inadequately treated mental health disorder - Suicidal or homicidal ideation - Liver measurements > 5x normal - International normalized ratio (INR) > 1.5 or platelet count <100k - Maintenance on methadone at doses of 30mg or greater - Allergy or sensitivity to study meds - Undergoing surgery during study - ongoing pain management with opioid - Currently pregnant, breastfeeding, or planning on conception - Unable to be safely injected with XR-NTX - Taken an investigational drug in another study in last 30 days - Treatment with XR-NTX for opioid or alcohol dependence in last 4 weeks - In jail, prison or pending legal action |
| Recruitment procedures | Participants were recruited from voluntary opioid treatment programs at eight treatment centers:   - 3 Western U.S. - 1 Midwest - 4 East | Participants were recruited from HIV treatment clinics at five sites:   - 1 Western U.S. - 2 Midwest - 2 East |
| N screened | 772 | 376 |
| N excluded | 202 Excluded:   - 82 dropped out before randomization - 61 did not meet criteria - 22 did not want to continue - 15 completed screening but were not eligible - 6 were eligible but not randomly assigned - 16 were excluded for other reasons | 262 Excluded:   - 87 had suppressed viral loads - 52 did not have OUD - 52 were lost to follow-up - 23 had a conflict with the study meds - 11 were HIV negative - 7 were excluded due to health risks - 30 were excluded for other reasons |
| Randomization | - 283 randomized to XR-NTX - 287 randomized to BUP-NX | - 55 were randomized to XR-NTX - 59 were randomized to TAU |
| Exclusions from current study | - None | - 8 TAU participants initiating methadone - 3 TAU participants only receiving oral naltrexone |

Table S2. Inverse probability of selection weight models

| Target population | Model |
| --- | --- |
| NSDUH | $\frac{P(CTN vs. NSDUH)}{P(CTN vs. NSDUH\vert Age,Sex,Race,Education,Employment, IDU, Depression, AUD, BUD)}$ |
| TEDS | $\frac{P(CTN vs. TEDS)}{P(CTN vs. TEDS\vert Age,Sex,Race,Education,Employment, IDU, Homelessness, AUD, BUD)}$ |
| ROI | $\frac{P(CTN vs. ROI)}{P(CTN vs. ROI\vert Age,Sex,Race,Education,Employment, IDU, Homelessness, AUD, BUD)}$ |

IDU = injection drug use, AUD = alcohol use disorder, BUD = benzodiazepine use disorder; NSDUH = National Survey on Drug Use and Health; TEDS = Treatment Episodes Dataset; ROI = Rural Opioids Initiative

Table S3. Distributions of inverse probability of selection weights and bias-variance tradeoff in weight trimming. Bias and precision of the stimulant use estimates are computed relative to untrimmed weights.

| Target population | Min | Mean | Max | Estimate | Bias | SE | Relative precision |
| --- | --- | --- | --- | --- | --- | --- | --- |
| Untrimmed | | | | | | | |
| NSDUH | 0.04 | 1.16 | 20.50 | -.303 |  | .228 |  |
| TEDS | 0.06 | 1.0 | 5.62 | -.319 |  | .175 |  |
| ROI | 0.26 | 0.98 | 8.81 | -.460 |  | .292 |  |
| Trimmed at the 1^st^ and 99^th^ percentiles^a^ | | | | | | | |
| NSDUH | 0.04 | 1.12 | 7.22 | -.309 | 0.006 | .224 | 1.018 |
| TEDS | 0.13 | 1.0 | 3.91 | -.323 | 0.004 | .174 | 1.006 |
| ROI | 0.27 | 0.96 | 5.89 | -.504 | 0.044 | .287 | 1.017 |
| Trimmed at the 2.5^th^ and 97.5^th^ percentiles^b^ | | | | | | | |
| NSDUH | 0.06 | 1.10 | 6.14 | -.322 | 0.019 | .223 | 1.022 |
| TEDS | 0.14 | 0.99 | 3.31 | -.325 | 0.006 | .175 | 1.000 |
| ROI | 0.27 | 0.93 | 3.70 | -.561 | 0.101 | .273 | 1.070 |

^a^Average bias = .0135, average relative precision gain = 1.007

^b^Average bias = .0297, average relative precision gain = 1.014

NSDUH = National Survey on Drug Use and Health; TEDS = Treatment Episodes Dataset; ROI = Rural Opioids Initiative
